# The Influence of Metabolic Syndrome and Sex on the DNA Methylome in Schizophrenia

**DOI:** 10.1155/2018/8076397

**Published:** 2018-04-03

**Authors:** Kyle J. Burghardt, Jacyln M. Goodrich, Brittany N. Lines, Vicki L. Ellingrod

**Affiliations:** ^1^Department of Pharmacy Practice, Wayne State University Eugene Applebaum College of Pharmacy and Health Sciences, 259 Mack Avenue, Suite 2190, Detroit, MI 48201, USA; ^2^Department of Environmental Health Sciences, University of Michigan School of Public Health, 1415 Washington Heights Ann Arbor, MI 48109, USA; ^3^Department of Clinical Social and Administrative Sciences, College of Pharmacy, University of Michigan, 428 Church Street, Ann Arbor, MI 48109, USA; ^4^Department of Psychiatry, School of Medicine, University of Michigan, 1301 Catherine, Ann Arbor, MI 48109, USA

## Abstract

**Introduction:**

The mechanism by which metabolic syndrome occurs in schizophrenia is not completely known; however, previous work suggests that changes in DNA methylation may be involved which is further influenced by sex. Within this study, the DNA methylome was profiled to identify altered methylation associated with metabolic syndrome in a schizophrenia population on atypical antipsychotics.

**Methods:**

Peripheral blood from schizophrenia subjects was utilized for DNA methylation analyses. Discovery analyses (*n* = 96) were performed using an epigenome-wide analysis on the Illumina HumanMethylation450K BeadChip based on metabolic syndrome diagnosis. A secondary discovery analysis was conducted based on sex. The top hits from the discovery analyses were assessed in an additional validation set (*n* = 166) using site-specific methylation pyrosequencing.

**Results:**

A significant increase in *CDH22* gene methylation in subjects with metabolic syndrome was identified in the overall sample. Additionally, differential methylation was found within the *MAP3K13* gene in females and the *CCDC8* gene within males. Significant differences in methylation were again observed for the *CDH22* and *MAP3K13* genes, but not *CCDC8*, in the validation sample set.

**Conclusions:**

This study provides preliminary evidence that DNA methylation may be associated with metabolic syndrome and sex in schizophrenia.

## 1. Introduction

Antipsychotics, in particular second-generation or atypical antipsychotics (AAPs), increase the risk of metabolic syndrome 2-3-fold in patients with schizophrenia due to their effects on weight and insulin resistance [1–4]. The metabolic syndrome consists of a cluster of metabolic disorders that include obesity, dyslipidemia, hypertension, and insulin resistance [5]. Together these risk factors are predictive of cardiovascular disease, type 2 diabetes, and mortality [6, 7]. Despite the risk of metabolic syndrome and other adverse events, AAPs provide many beneficial, therapeutic benefits. Increased awareness including enhanced metabolic monitoring and pharmacologic treatment of metabolic disorders that arise during AAP use (e.g., blood pressure medication use) has helped to lower the risk of metabolic syndrome, yet this has not completely removed it. Additionally, the use of AAPs has expanded from schizophrenia to other populations such as pediatric which are particularly sensitive to these metabolic effects [8–10]. Therefore, a better understanding of the molecular mechanisms underlying metabolic syndrome in patients with schizophrenia is necessary so that personalized interventions and/or newer therapies can be developed that will minimize or remove the risk.

Previous work has linked aberrant genetic regulation of the folate cycle to metabolic syndrome risk in schizophrenia patients treated with AAPs [11]. Additionally, treatment of schizophrenia patients with folate may aid in improving some aspects of metabolic syndrome [12]. These findings suggest that a properly functioning folate system may be important to minimizing the risk for AAP-induced metabolic syndrome. Dysfunctional folate regulation could be causing metabolic syndrome for several reasons [13, 14]. At the molecular level, a product of the folate cycle is methyl molecules used for various cellular reactions including lipid, protein, and DNA methylation. Thus, it has been suggested that altered gene regulation through changes to DNA methylation may be responsible for AAP-associated metabolic syndrome.

DNA methylation at the global level may be altered in schizophrenia patients with metabolic syndrome in sex-specific ways [15]. Despite this previously identified association between global DNA methylation and AAP-induced metabolic syndrome, only one study has examined gene-specific methylation at the catechol-O-methyltransferase *(COMT)* gene and reported a negative finding [16]. Within the current study, we used an epigenome-wide strategy to identify and potentially validate candidate genes or regions that are associated with metabolic syndrome in a cohort of well-characterized schizophrenia subjects while also determining any sex-specific effects that may be present.

## 2. Methods

### 2.1. Subject Population Recruitment and Assessment

Potential subjects were recruited from mental health clinics and with public advertisements in the Southeastern Michigan and neighboring areas. A preliminary phone screening was used to assess for the following inclusion criteria: Diagnostic and Statistical Manual IV diagnosis with a schizophrenia-spectrum disorder, age 18 to 90 years, presently treated with an antipsychotic medication with no dosage changes in the past 6 months, and no known metabolic diseases such as dyslipidemia, hypertension, or diabetes prior to starting their antipsychotic treatment. Subjects were excluded if they were pregnant or unable to give blood. Potential subjects interested in participating were invited to the Michigan Clinical Research Center (MCRC) which is supported by the Michigan Institute for Clinical and Translational Research (MiCHR) to undergo full informed consent as approved by the University of Michigan Institutional Review Board. The study was registered with ClinicalTrials.gov (NCT00815854).

Following consent, subjects underwent a medical and medication history questionnaire which captured current and past medication use. Pharmacy and clinical records were used to verify dosages. For the purposes of description, antipsychotics were grouped according to their potential to cause metabolic side effects (i.e., high versus medium versus low) [17, 18]. Subjects on olanzapine and clozapine were placed in the high-risk metabolic group; subjects on quetiapine, paliperidone, and risperidone were placed in the medium-risk group; and subjects on aripiprazole and ziprasidone were placed in the low-risk group. We have employed this empirical categorization in previous metabolic studies [19]. Subjects underwent psychiatric screening by a trained clinical research assistant using the Structure Clinical Interview for DSM diagnoses (SCID-4) in order to confirm the diagnosis of schizophrenia [20]. Vital signs and anthropometric data including weight, height, hip circumference, and waist circumference were assessed by clinical research center nursing staff. All subjects underwent a fasting blood draw that was used for laboratory analyses (glucose and lipid panels) and genomic DNA extraction. Glucose and lipid levels were analyzed by the University of Michigan Hospital System (UMHS) laboratories. Samples for both the discovery and validation groups described in the results were collected using the above inclusion/exclusion criteria and protocol. Ninety-six samples were chosen for the discovery analysis. The discovery group was selected to include one-half with metabolic syndrome equally matched based on age, race, and sex (e.g., 48 subjects with metabolic syndrome matched with 48 subjects without metabolic syndrome). The remainder of the recruited subjects (166 additional samples) were used for validation of the discovery findings.

### 2.2. Genetics Analysis: Extraction and Preparation of Genomic DNA

Genomic DNA was extracted by the salt precipitation method [21] and cleaned using commercially available kits. DNA was quantified on a Qubit fluorimeter (Life Technologies) and 1 *μ*g of bisulfite was converted using the Zymo EZ DNA Methylation-Gold kit (Zymo Technologies) according to manufacturer specifications.

### 2.3. Genetics Analysis: Discovery Analysis

For the discovery analysis, converted samples were submitted to the University of Michigan DNA Sequencing Core for analysis on the Illumina HumanMethylation450 BeadChip (“450K”). The core returned raw IDAT files for subsequent processing and statistical analysis by investigators. Discovery analyses were conducted in the combined samples (96 subjects) and within each sex (49 male, 47 female).

### 2.4. Genetics Analysis: Validation Analysis

For the validation analyses, primer sets were chosen based on the discovery findings where the goal was to choose locations within the same CpG island or the nearest CpG island to the top discovery finding for the combined analysis and the sex-specific analyses. Site-specific methylation was analyzed by the method of pyrosequencing on a PyroMark MD 96. Commercially available primer sets from Qiagen (Redwood City, CA, USA) were utilized for the *CDH22* (in a nearby CpG island ~300 base pairs away) and *CCDC8* genes (same CpG island). The *CDH22* primer set was designed to obtain an amplicon of approximately 115 base pairs that would analyze methylation in chromosome 20 at positions 44880277, 44880264, and 44880250 following pyrosequencing (genomic coordinates using GRCh37/hg19). The *CCDC8* primer set was designed to obtain an amplicon of 189 base pairs and analyze methylation at four chromosome 19 locations (46915716, 46915706, 46915704, and 46915701). Finally, a primer set for the *MAP3K13* gene was designed using the Qiagen Assay Design 2.0 program (in the same CpG island as the discovery finding). The resultant primers amplified a 146-base-pair region in the *MAP3K13* gene on Chromosome 3 for methylation analysis at positions 185000790, 185000779, 185000774, and 185000760. Primers for the self-designed *MAP3K13* assay are available upon request. All samples were performed in triplicate, and each batch was normalized by constructing a standard curve with samples of known methylation to account for bisulfite PCR bias [22]. No samples were removed due to excess variation amongst the replicates (defined as a coefficient of variation > 5%).

### 2.5. Statistical Analysis

Values are reported in mean ± standard deviations (s.d.). Student *t*-, chi-square, or Fisher's exact test was used for comparison of demographic and clinical variables between metabolic syndrome groups and discovery and validation groups. The epigenome-wide analysis for the discovery of differentially methylated genes associated with metabolic syndrome employed the use of RnBeads [23]. RnBeads is a comprehensive R statistical software package that enables users to utilize specific workflows for processing, normalizing, and analyzing DNA methylation data from the Illumina HumanMethylation450 BeadChip. Within RnBeads, our obtained data was loaded in raw IDAT file form where it was preprocessed and normalized according to published biostatistical and bioinformatics workflows which included correction for color bias, quantile normalization, probe-type bias, and batch adjustment [24, 25]. Preprocessed and normalized *M* values were then analyzed by CpG site (overall sample) or CpG island (sex-specific analysis) using linear regression with the *limma* package [26]. The CpG island analysis, employed through the RnBeads package in R, uses annotation data to group probes within the same CpG island and constructs a “combined” *p* value to assess that CpG island's overall association with metabolic syndrome by regression with the *limma* package. Three linear regressions were performed for discovery of differential methylation based on metabolic syndrome: (1) CpG sites (total of 393,193 sites) in the overall discovery sample, (2) CpG islands (total of 25,352 CpG islands) in males within the discovery samples, and (3) CpG islands in females within the discovery sample. Each regression used metabolic syndrome as the independent variable of interest while adjusting for other relevant variables. For the CpG site analysis in the overall discovery sample, regressions were adjusted for smoking status, antipsychotic type, and estimated cell types using the Houseman et al. method in R [27]. For the regional CpG island analyses based on sex, both the male and female regressions used age, smoking status, antipsychotic type, and estimated cell-type compositions. All regressions used the *sva* package within RnBeads to detect batch effects and control them by adding estimated surrogate variables as covariates to each model [28]. Top differentially methylated CpG sites or CpG islands were corrected for multiple testing using a false discovery rate (FDR, *q* value) cutoff of less than 0.05. [29].

Validation analyses were conducted in a separate sample of subjects to potentially replicate the top differentially methylated findings from the overall and sex-specific discovery analyses. Validation analyses used linear regressions, in a similar format to the epigenome-wide analysis, where each methylation site (within the three genes assessed) served as the dependent variable and metabolic syndrome status served as the independent variable of interest while adjusting for age, sex, race, smoking status, and antipsychotic type. A *p* value < 0.05 was considered statistically significant for the validation analyses.

### 2.6. Pathway Analysis

An exploratory pathway analysis utilizing the discovery analysis data was performed with Ingenuity Pathway Analysis (IPA) software build version: 430520M, content version: 31813283, release date: December 5, 2016 (Qiagen, Redwood City, CA, USA). Such an analysis may reveal pathway or network perturbations not captured by the top CpG site or CpG island approach employed in the discovery analysis due to various reasons including a lack of power. For the overall discovery analysis, the top 100 differentially methylated genes corresponding to the CpG sites were analyzed in IPA. For the sex-specific discovery analyses, the top 50 CpG islands genes were entered into IPA for analysis. The top 100 or 50 genes were chosen as an arbitrary cutoff that would include a representation of potentially influenced pathways by metabolic syndrome in the overall and sex-specific analyses. Alternate gene sets (e.g., top 1000) in the pathway analysis did not result in the identification of other pathways (data not shown). The IPA reference set chosen was the Ingenuity Knowledge Base, and the findings were restricted to humans only in the Core Analysis module. Top canonical pathways with an FDR-corrected *p* value below 0.05 were considered statistically significant for each analysis.

## 3. Results

### 3.1. Discovery and Validation Group Characteristics

The discovery sample, consisting of a total of 96 subjects, had an average age of 49.5 ± 8.4 years, 51% were male, 60% were Caucasian, and 35% were African-American. The distribution of antipsychotic type was similar between discovery and validation groups. As designed, approximately 50% of the discovery sample had a diagnosis of metabolic syndrome matched for age and race and split evenly for sex. There was a trend for a lower rate of smoking in females when compared to males (*p* = 0.1) in the discovery sample. The validation sample had a total of 166 subjects. The average age of the validation sample was 43.9 ± 12.0 years, 64% were male, and 46% had metabolic syndrome. The discovery and validation groups were similar except for a non-significant trend for more males in the validation group (*p* = 0.08). Both samples' demographic and clinical variables can be found in Table 1. Additionally, a breakdown by sex for each sample can be found in Table 2. Significant differences were not noted between males and females in the validation group.

### 3.2. Discovery: Differentially Methylated Sites Based on Metabolic Syndrome

As described in Methods, the included covariates in the final model to estimate the top differentially methylated CpG sites based on metabolic syndrome in the overall discovery sample were smoking status, antipsychotic type, and cell composition. A *Q*-*Q* plot with inflation factor was used to characterize the *p* value distributions and estimate the appropriateness of the model. We compared the model with and without the correction for batch effects through the addition of estimated surrogate variables with RnBeads. The comparisons can be found in Supplemental Figure 1. The model without batch effect correction had an inflation factor of 0.978 while correction for batch effects improved the model's visual fit and the associated inflation factor to 1.006. This suggests that there were small, but still present, sources of additional variation that were unaccounted for by our included covariates. From the *Q*-*Q* plot, the associations of methylation with respect to metabolic syndrome deviate from the null at higher *p* values as would be expected.  Table 3 shows the top differentially methylated CpG sites associated with metabolic syndrome at a FDR less than 0.1. The top five CpG sites met a predefined FDR cutoff of <0.05 and were found in the following genes: cadherin-like 22 *(CDH22)*, family with sequence similarity 19 (chemokine- (C-C motif-) like), member A2 *(FAM19A2)*, cadherin-like 22 *(CDH5)*, casein kinase 1 *(CSNK1E)*, and Delta/notch-like EGF repeat *(DNER)*. An expanded table with proposed biological functions as well as previous correlations from the literature for each site's corresponding gene can be found in Supplementary Table 1. For further exploration, the top 100 differentially methylated CpG sites based on metabolic syndrome in the overall discovery sample can be found in Supplementary Table 2.

### 3.3. Discovery: Sex-Specific Differential Methylation in Metabolic Syndrome

Following our analyses in the overall discovery sample, we conducted a sex-specific analysis of differential methylation based on metabolic syndrome. For this secondary analysis, we chose to conduct analyses at the regional level of CpG islands. This was done to increase power in a limited sample size by decreasing the number of statistical tests being conducted.

For the male population, the model to identify top differentially methylated CpG islands based on metabolic syndrome included the following covariables: age, smoking status, antipsychotic type, and cell-type composition. The model that included the estimated surrogate variables had an improved lambda based on a *Q*-*Q* plot (lambda without surrogate variables = 0.889 versus lambda with surrogate variables = 1.001). The CpG islands associated with metabolic syndrome in males with an FDR *p* value < 0.1 can be found in Table 4. The top result, in the coiled-coil domain containing 8 *(CCDC8)* gene, was statistically significant after FDR correction.

We performed the same analysis using the same regression variables in females. Including the surrogate variables in the model improved the lambda from 0.893 to 1.021. Table 4 also contains the female analysis results. The top two CpG islands, found in the mitogen-activated protein kinase kinase kinase 13 *(MAP3K13)* and transmembrane phosphoinositide 3-phosphatase and tensin homolog 2 *(TPTE2)* genes, were statistically significant after FDR correction. An expanded table, for further exploration, showing the top 50 CpG islands for each sex can be found in Supplementary Table 3.

### 3.4. Discovery: Pathway Analysis

The exploratory pathway analysis of the overall sample results revealed that differential methylation related to metabolic syndrome in schizophrenia was enriched in the Wnt/*β*-catenin signaling pathway (FDR *p* value = 6.21 × 10^−4^). For the analysis in females only, the axonal guidance signaling pathway was the most enriched pathway (FDR *p* value = 6.22 × 10^−4^). Finally, the FAK signaling pathway was the top pathway for the male analysis of CpG islands associated with metabolic syndrome (FDR *p* value = 1.32 × 10^−4^). The top ten pathways for each analysis along with the identified genes that caused enrichment can be found in Supplementary Table 4.

### 3.5. Validation Analyses of Top Differentially Methylated Genes from Discovery

In the absence of access to a larger sample set for the discovery analyses, we sought to validate the top discovery findings in an additional sample of schizophrenia subjects from the same recruitment pool. For validation, methylation was analyzed by site-specific pyrosequencing at three sites: (1) the top differentially methylated CpG site in the overall discovery sample *(CDH22)* and (2) the top differentially methylated CpG islands for males *(CCDC8)* and (3) females *(MAP3K13)*. Significant associations at Chr20:44880277 and Chr20:44880264 in the *CDH22* gene were identified in the overall sample which held when adjusting for age, race, gender, smoking, and antipsychotic type (both *p* = 0.04). At both sites, higher methylation (hypermethylation) was observed in subjects with metabolic syndrome. The third site Chr20:44880250, assessed in the *CDH22* gene, did not reach statistical significance (*p* = 0.7).

Three sites within the *MAP3K13* gene at genomic locations Chr3:185000779, Chr3:185000774, and Chr3:185000760 were associated with metabolic syndrome status in females after adjusting for age, race, smoking status, and antipsychotic type (*p* = 0.01, 0.04, and 0.01, resp.). Consistent with the discovery analysis, hypomethylation of *MAP3K13* was seen in female subjects with metabolic syndrome. *CCDC8* methylation did not show significant differences based on metabolic syndrome within males. The details of the unadjusted and adjusted validation analyses are found in Table 5.

## 4. Discussion

The purpose of this study was to identify areas of the DNA methylome that may be altered in subjects with AAP-associated metabolic syndrome [15]. To this end, we identified overall and sex-specific differentially methylated genes in a discovery sample of 96 schizophrenia subjects. Two of the three findings from the discovery group were validated in an additional group of schizophrenia subjects.

### 4.1. Differentially Methylated Genes Associated with Metabolic Syndrome

Differentially methylated CpG sites within the overall discovery sample were located within genes with either a known biological function in a cardiometabolic illness and/or a previously reported association with a metabolic phenotype or disease (Supplementary Table 1). The top site, located in the cadherin-like 22 *(CDH22)* gene, had increased methylation in subjects with metabolic syndrome (i.e., hypermethylation). A member of the cadherin superfamily, this gene codes for a cell-adhesion protein that is predominately expressed in the brain and is important for tissue development and morphogenesis. This gene has been associated with type 2 diabetes in a previous genetic variation study [30]. Within this study, out of the top 5 most significant single nucleotide polymorphisms associated with type 2 diabetes, 3 were found in *CDH22*. It should be noted that within this study, this finding was not replicated in a separate data set. Nevertheless, it may be that *CDH22* regulation, through both genetic and epigenetic mechanisms, could point to a potentially important role for this gene in metabolic disease. Additionally, differential methylation was identified in the cadherin 5, type 2 *(CDH5)* gene, which is also in the cadherin superfamily. This particular cadherin isoform is highly important in the development of vascular endothelium which our group has shown to be influenced by AAP use, folate metabolism, and genetic variation [11, 12, 31]. Specifically, we previously have shown that genetic variation in the rate-limiting enzyme in folate metabolism, methylenetetrahydrofolate *(MTHFR)*, as well as endothelial nitric oxide synthetase *(eNOS)* is associated with a greater risk for endothelial dysfunction, a predictor of cardiovascular morbidity and mortality. Additional work may be needed to understand if genetic regulation at *CDH5* confers additional risk. Altogether, these findings may add further evidence of the complex links between altered folate regulation, DNA methylation, and AAP-associated metabolic syndrome and cardiovascular disease.

Within the top differentially methylated CpG sites in the overall population, several genes related to protein regulation and function were present (e.g., *CSNK1E*, *E2F2*, *PSMB8*, *PPP1R12B*, and *PDK1*). Control of protein function and action through phosphorylation and other modifications play a central role in several disease states including diabetes, lipid metabolism, insulin resistance, and metabolic syndrome [32, 33]. Additionally, altered basal and insulin-stimulated protein phosphorylation has been identified with AAP treatment in both preclinical models and patients [34–36]. In particular, the *CSNK1E* gene has been linked to the pathophysiology of schizophrenia and bipolar disorder which are the main conditions for which antipsychotics are used [37, 38]. Further work incorporating the power of DNA methylomics and proteomics in AAP treatment may yield further insight into psychiatric disease and its treatment.

### 4.2. Sex-Specific Methylation in Metabolic Syndrome: Females

In addition to looking at the associations between the DNA methylome and metabolic syndrome in schizophrenia in an overall manner, region-specific DNA methylation, at the CpG Island level, was performed within each sex based on our previous findings suggesting that sex may play a role [15]. The top differentially methylated CpG island associated with metabolic syndrome in females was in the gene encoding for the mitogen-activated protein kinase kinase kinase 13 *(MAP3K13)* protein. This protein, a member of the serine/threonine phosphatase kinase family, interacts with and regulates other proteins through its ability to phosphorylate specific mitogen-activated proteins including MAP2K7/MKK7 and MAPK8/JNK [39, 40]. Epigenetic regulation of this protein may play a role in MAPK and Jun amino terminal kinase (JNK) signaling pathways, both shown to play an important role in glucose homeostasis, a defining feature of the metabolic syndrome [41–43]. Overall, female subjects with metabolic syndrome had lower methylation in the investigated *MAP3K13* CpG island (both in the discovery and validation analyses) compared to female subjects without metabolic syndrome which may suggest higher expression and possibly activity of the kinase. Further work is needed to understand the effect of epigenetic regulation on *MAP3K13* expression and activity.

### 4.3. Sex-Specific Methylation in Metabolic Syndrome: Males

The top differentially methylated CpG island associated with metabolic syndrome in males was found in the coiled-coil domain containing 8 *(CCDC8)* gene, although this finding was not replicated in our validation sample. The *CCDC8* gene (alias name protein phosphatase 1, regulatory subunit 20 (PPP1R20)) encodes a protein involved in cell apoptosis following DNA damage as well as human growth and development and genomic integrity [44, 45]. Notably, this protein has been shown to modulate alternative splicing of the insulin receptor (INSR) [46], which may have downstream effects on MAPK/AKT signaling. Again, given that insulin resistance is a key feature of metabolic syndrome, further work with this gene and its effects on the MAPK/AKT pathway may be warranted.

### 4.4. Exploratory Pathway Analyses

The Wnt/*β*-catenin pathway, the most enriched pathway in the overall analysis, involves molecules from other pathways to regulate cell-specific processes including fate, proliferation, and migration. This pathway has been linked to insulin signaling and sensitivity and lipid metabolism which all have been known to be influenced by AAP treatment [47–52]. For females, the top canonical pathway was the axonal guidance signaling pathway which is involved in determining how nervous system axons reach their target. Such a pathway may be of importance in a psychiatric disorder where significant overlap is seen in metabolic and nervous system processes when considering both the disease itself as well as the medications used to treat the symptoms. Interactions between these pathways have been identified in other models of metabolic disease [53]. The FAK signaling pathways were the most enriched pathways in the male analysis. This pathway is involved in cell movement and adhesion and has also been linked to glucose dysregulation and insulin signaling [54, 55]. In summary, pathway analyses revealed sex-specific enriched pathways when considering DNA methylation in the context of metabolic syndrome. Despite the differences, an underlying theme of involvement in insulin signaling was present in the identified pathways (see Supplementary Table 4).

### 4.5. Strengths and Limitations

The current study utilized a sample of schizophrenia subjects who were stable in their antipsychotic therapy for 6 or more months to identify DNA methylation changes associated with metabolic syndrome. The sample was well characterized using detailed medication histories as well as anthropometric and metabolic assessments to diagnose metabolic syndrome. Some limitations should be considered when interpreting the findings of this study. DNA methylation was assessed in peripheral blood which is composed of multiple cell types. While we could use statistical techniques to estimate cell-type composition and control for it in the discovery analyses, we did not have access to cell-type composition in the gene-specific validation analyses. Validation analyses occurred by choosing methylation sites within the same CpG island as the discovery sites or, for the *CDH22* validation, the nearest CpG island which was within 300 base pairs of the original discovery site. We chose to focus validation work within CpG islands due to their known importance in gene regulation. Other sites may have stronger associations with metabolic syndrome, or in the case of the *CCDC8* gene which was not validated, other areas of the gene may have stronger associations with metabolic syndrome. Deeper, gene-specific methylation profiling within a tissue of interest (e.g., adipose, muscle, or brain) in various models should be considered to further understand the role of epigenetics in antipsychotic-induced metabolic syndrome. We did not have access to RNA samples to assess gene expression levels. Future work will need to functionally validate the effect of methylation on gene expression or other downstream products of the gene(s). Based on previous work establishing sample sizes and effect sizes in epigenome-wide studies, the discovery sample size was limited in its ability to detect smaller changes (e.g., smaller effect sizes) in methylation; however, DNA methylation at two of our genes was validated in an additional sample of schizophrenia subjects which does strengthen the findings in its present form [56]. The study includes a population on various AAPs. For the purposes of our study, we were interested in capturing a population on any AAP since all AAP increases cause weight gain and increase the risk of metabolic syndrome [57]. Our analyses (not shown) did not identify significant effects of AAP dosage; however, these analyses may have been underpowered to allow for appropriate interpretation. Future work may need to begin to analyze specific antipsychotics in mechanistic studies at specified dosages to better design interventions that prevent this side effect. Finally, this study utilized cross-sectional samples without a healthy control group. This makes determining cause and effect (e.g., if the medications are inducing changes in DNA methylation which subsequently causes metabolic syndrome or vice versa) and the effect of the psychiatric disease itself difficult. Evidence exists suggesting AAP effects on molecular features may be specific to specific psychiatric illnesses [58]. Nevertheless, the identification of gene methylation associated with AAP-associated metabolic syndrome will serve to direct further studies that look at DNA methylation changes before and after antipsychotic treatment coupled with a healthy control group to assess causation between metabolic syndrome and gene methylation.

## 5. Conclusion

Within our study, we identified gene methylation changes that are associated with antipsychotic-associated metabolic syndrome and changes that are specific to sex. The results here are preliminary, and future work is needed to understand the mechanistic role of these gene changes and possible therapies that could target and potentially prevent the negative consequences of antipsychotic-induced metabolic syndrome.

## Figures and Tables

**Table 1:** Demographic and clinical characteristics of discovery and validation groups.

	Discovery group (*n* = 96)	Validation group (*n* = 166)
Age (years ± s.d.)	49.8 ± 7.4	43.9 ± 12.0
Sex (% male)	51	64
Caucasian (%)/African-American (%)	60/35	53/32
Metabolic syndrome (%)	50	46
% currently smoking	50	51
Olanzapine/clozapine (%)	29	29
Quetiapine/paliperidone/risperidone (%)	38	39
Aripiprazole/ziprasidone (%)	33	32

The table depicts the mean ± s.d. or % values for the discovery and validation groups. No statistically significant differences were noted between the groups. A nonsignificant trend for more males in the validation group was observed (*p* = 0.08).

**Table 2:** Discovery and validation group broken down by sex.

	Discovery group		Validation group	
	Males (*n* = 49)	Females (*n* = 47)	Males (*n* = 100)	Females (*n* = 66)
Age (years ± s.d.)	49.4 ± 8.64	49.7 ± 8.29	42.9 ± 11.4	45.7 ± 13.1
Caucasian (%)/African-American (%)	55/40	65/30	53/36	53/25
Metabolic syndrome (%)	50	51	44	51
% currently smoking	57	43	55	56
Olanzapine/clozapine (%)	25	24	31	26
Quetiapine/paliperidone/risperidone (%)	35	38	44	31
Aripiprazole/ziprasidone (%)	40	38	25	43

The table depicts the mean ± s.d. or % values for the discovery and validation groups. No statistically significant differences were noted between males and females for either group. There was a trend for decreased smoking in females in the discovery group (*p* = 0.1).

**Table 3:** Top differentially methylated sites based on metabolic syndrome.

CpG probe ID	Gene	Chromosome	Position	CpG type	Fold change^a^	Raw *p* value	FDR-corrected *p* value
cg04640913	Cadherin-like 22 *(CDH22)*	20	44880515	South shore	0.123	9.26 × 10^−07^	0.02^∗^
cg12501957	Family with sequence similarity 19 (chemokine- (C-C motif-) like), member A2 *(FAM19A2)*	12	62629234	Open sea	−0.0266	1.05 × 10^−06^	0.04^∗^
cg05086443	Cadherin-like 22 *(CDH5)*	16	66437349	South shore	−0.0215	3.45 × 10^−06^	0.04^∗^
cg16653173	Casein kinase 1 *(CSNK1E)*	22	38713453	South shore	0.0675	3.83 × 10^−06^	0.04^∗^
cg16656316	Delta/notch-like EGF repeat *(DNER)*	2	230280621	Open sea	−0.0764	7.85 × 10^−06^	0.04^∗^
cg06378976	Transcription factor EB *(TFEB)*	6	41703613	South shore	0.135	1.05 × 10^−05^	0.08
cg04457354	E2F transcription factor 3 *(E2F2)*	6	20447442	Open sea	−0.0221	2.03 × 10^−05^	0.08
cg04953503	Melanophilin *(MLPH)*	2	238420656	Open sea	−0.00763	2.24 × 10^−05^	0.08
Cg05434957	Islet autoantigen 1 *(ICA1)*	7	8301435	Island	0.118	2.26 × 10^−05^	0.09
cg08464505	ATPase, class VI, type 11A *(ATP11A)*	13	113425982	South shore	−0.0113	2.87 × 10^−05^	0.09
Cg22158175	Proteosome subunit, beta type, 8 *(PSMB8)*	6	32809475	North sea	−0.0162	3.10 × 10^−05^	0.09
Cg17492940	Protein phosphatase 1, regulatory subunit 12B *(PPP1R12B)*	1	202407102	Open sea	−0.00380	3.30 × 10^−05^	0.1
Cg04033559	Pyruvate dehydrogenase kinase, isozyme 1 *(PDK1)*	2	173461819	Open sea	−0.312	3.46 × 10^−05^	0.1

Top differentially methylated sites based on metabolic syndrome status with an FDR *p* value < 0.1. Only FDR < 0.05 was considered statistically significant in this study. Columns 1 and 2 give the probe ID and associated gene name. Columns 3 and 4 give the genomic location (GRCh37/hg19) of the CpG site and CpG classification of the probe with respect to CpG islands (i.e., island versus shore versus sea). The final columns give the fold change with direction, unadjusted, and FDR-corrected *p* values. ^a^Fold change calculated by log2 of the quotient in methylation in subjects with metabolic syndrome compared to subjects without metabolic syndrome. Positive fold change indicates an increase in methylation (hypermethylation) in the metabolic syndrome group. ∗ indicates statistical significance based on an FDR cutoff below 0.05

**Table 4:** Top differentially methylated CpG islands based on metabolic syndrome in males and females.

Female						Male					
Chromosomal location (Chr:region)	Number of CpGs in island	Gene name	Fold change^a^	Raw *p* value	FDR-corrected *p* value	Chromosomal location (Chr:region)	Number of CpGs in island	Gene name	Fold change^a^	Raw *p* value	FDR-corrected *p* value
3: 185000558–185000896	33	Mitogen-activated protein kinase kinase kinase 13 *(MAP3K13)*	−0.196	0.000936	0.0423^∗^	19: 46915312–46915802	44	Coiled-coil domain containing 8 *(CCDC8)*	0.0930	0.0000186	0.0325^∗^
13: 20135400–20136041	53	Transmembrane phosphoinositide 3-phosphatase and tensin homolog 2 *(TPTE2)*	0.0267	0.00101	0.0423^∗^	17: 40558006–40558274	19	Polymerase I and transcript release factor *(PTRF)*	−0.129	0.000278	0.102

Shows the CpG islands for the sex-specific methylation discovery analysis with an FDR < 0.1. FDR < 0.05 was considered significant. Column 1 provides the genomic location of the CpG island (GRCh37/hg19), column 2 provides the number of CpG sites located within the island according to annotation data, and column 3 provides the gene name where the CpG island is found. The remaining columns provide the fold change with direction, unadjusted, and FDR-corrected *p* values. ^a^Fold change calculated by log2 of the quotient in methylation in subjects with metabolic syndrome compared to subjects without metabolic syndrome. Positive fold change indicates an increase in methylation (hypermethylation) in the metabolic syndrome group. ∗ indicates statistical significance based on an FDR cutoff below 0.05

**Table 5:** Site-specific validation methylation analyses based on discovery findings.

Metabolic syndrome status		Validation population: males and females (*n* = 166)			Validation population: males only (*n* = 100)				Validation population: females only (*n* = 66)			
		*CDH22* Chr20:44880277	*CDH22* Chr20:44880264	*CDH22* Chr20:44880250	*CCDC8* Chr19:46915716	*CCDC8* Chr19:46915706	*CCDC8* Chr19:46915704	*CCDC8* Chr19:46915701	*MAP3K13* Chr3:185000790	*MAP3K13* Chr3:185000779	*MAP3K13* Chr3:185000774	*MAP3K13* Chr3:185000760
Metabolic syndrome^a^	Crude beta ± s.e. (*p* value)	1.034 ± 0.520 (*p* = 0.0488^∗^)	1.14 ± 0.508 (*p* = 0.264)	0.334 ± 0.488 (*p* = 0.5)	−0.149 ± 1.09 (*p* = 0.9)	0.200 ± 1.18 (*p* = 0.9)	0.355 ± 1.03 (*p* = 0.7)	−0.063 ± 1.09 (*p* = 0.9)	−0.584 ± 0.491 (*p* = 0.2)	−0.969 ± 0.367 (*p* = 0.0110^∗^)	−0.705 ± 0.481 (*p* = 0.1)	−0.696 ± 0.517 (*p* = 0.2)
	Adjusted beta ± s.e.	1.27 ± 0.531 (*p* = 0.0469^∗^)	1.29 ± 0.625 (*p* = 0.0414^∗^)	0.222 ± 0.646 (*p* = 0.7)	0.235 ± 1.20 (*p* = 0.8)	0.573 ± 1.31 (*p* = 0.7)	0.547 ± 1.12 (*p* = 0.6)	0.711 ± 1.15 (*p* = 0.5)	−0.258 ± 0.575 (*p* = 0.7)	−1.12 ± 0.437 (*p* = 0.0137^∗^)	−1.17 ± 0.578 (*p* = 0.0491^∗^)	−1.42 ± 0.583 (*p* = 0.0192^∗^)

Gives beta values with standard error for unadjusted and adjusted regression model with methylation site as dependent variable and metabolic syndrome status as the independent variable. CDH22 validation regressions performed in overall validation population, CCDC8 validation regressions performed in males only, and MAP3K13 validation regressions performed in females only. CDH22 regressions adjusted for age, gender, race, smoking status, and antipsychotic type. CCDC8 and MAP3K13 regressions adjusted for age, race, smoking status, and antipsychotic type. ^a^Reference group is subjects without metabolic syndrome; therefore, a positive beta value indicates hypermethylation in subjects with metabolic syndrome while a negative beta value indicates hypomethylation in subjects with metabolic syndrome. ∗ indicates statistical significance based on a *p* value below 0.05.

## Supplementary Materials

Supplementary Materials Supplementary Figure 1: *Q*-*Q* plot of overall discovery sample epigenome-wide analysis. *Q*-*Q* plots for the model assessing the association between metabolic syndrome and methylation site using the Illumina HumanMethylation450 BeadChip for the overall population. Both models included smoking status, antipsychotic type, CD4T, CD8T, granulocytes, monocytes, and natural killer cell counts as covariates. Plot (a) depicts the *Q*-*Q* plot before performing surrogate variable adjustment (lambda = 0.978) and (b) after performing surrogate variable adjustment (lambda = 1.0006). Supplementary Table 1: annotated top differentially methylated CpG sites (FDR < 0.1) associated with metabolic syndrome in the overall sample. Top differentially methylated sites (FDR < 0.1) associated with metabolic syndrome annotated with biological function and previous links to cardiometabolic phenotypes in the literature. It expands Table 1 from the main manuscript including a description of the known or proposed biological function of the associated gene and previous literature references investigating its role in cardiometabolic outcomes (references listed at end of the supplementary file). Supplementary Table 2: top 100 differentially methylated CpG sites associated with metabolic syndrome in the overall sample. Top 100 CpG results with annotated genes from linear regression of methylation sites based on metabolic syndrome adjusted for smoking status, antipsychotic type, estimated cell types, and batch effects (components estimated using the *sva* package) in the overall sample. Supplementary Table 3: top 50 differentially methylated CpG islands associated with metabolic syndrome from the sex-specific analysis. Top 50 annotated results from sex-specific linear regression of CpG islands based on metabolic syndrome adjusted for smoking status, antipsychotic type, estimated cell types, and batch effects (components estimated using the *sva* package). Supplementary Table 4: top 10 enriched pathways for each discovery analysis. The 1000 CpG sites (or CpG islands for sex-specific analysis) with the smallest *p* values from the discovery analyses were entered into the Core Analysis module of Ingenuity Pathway Analysis (IPA) software. The top 10 canonical pathways are listed in the table for each analysis along with FDR-correct *p* values, ratios, and genes.
