# Supplementary material for: The Influence of Metabolic Syndrome and Sex on the DNA Methylome in Schizophrenia
Source: Int J Genomics. 2018 Apr 3;2018:8076397. doi: 10.1155/2018/8076397 (PMC5903198; doi:10.1155/2018/8076397)
Supplement: Supplementary Materials — Supplementary Figure 1: Q-Q plot of overall discovery sample epigenome-wide analysis. Q-Q plots for the model assessing the association between metabolic syndrome and methylation site using the Illumina HumanMethylation450 BeadChip for the overall population. Both models included smoking status, antipsychotic type, CD4T, CD8T, granulocytes, monocytes, and natural killer cell counts as covariates. Plot (a) depicts the Q-Q plot before performing surrogate variable adjustment (lambda = 0.978) and (b) after performing surrogate variable adjustment (lambda = 1.0006). Supplementary Table 1: annotated top differentially methylated CpG sites (FDR < 0.1) associated with metabolic syndrome in the overall sample. Top differentially methylated sites (FDR < 0.1) associated with metabolic syndrome annotated with biological function and previous links to cardiometabolic phenotypes in the literature. It expands Table 1 from the main manuscript including a description of the known or proposed biological function of the associated gene and previous literature references investigating its role in cardiometabolic outcomes (references listed at end of the supplementary file). Supplementary Table 2: top 100 differentially methylated CpG sites associated with metabolic syndrome in the overall sample. Top 100 CpG results with annotated genes from linear regression of methylation sites based on metabolic syndrome adjusted for smoking status, antipsychotic type, estimated cell types, and batch effects (components estimated using the sva package) in the overall sample. Supplementary Table 3: top 50 differentially methylated CpG islands associated with metabolic syndrome from the sex-specific analysis. Top 50 annotated results from sex-specific linear regression of CpG islands based on metabolic syndrome adjusted for smoking status, antipsychotic type, estimated cell types, and batch effects (components estimated using the sva package). Supplementary Table 4: top 10 enriched pathways for each [file 8076397.f1.docx]

# Supplementary Files

**Figure 1. QQ plot of Overall Discovery Sample Epigenome-wide Analysis**


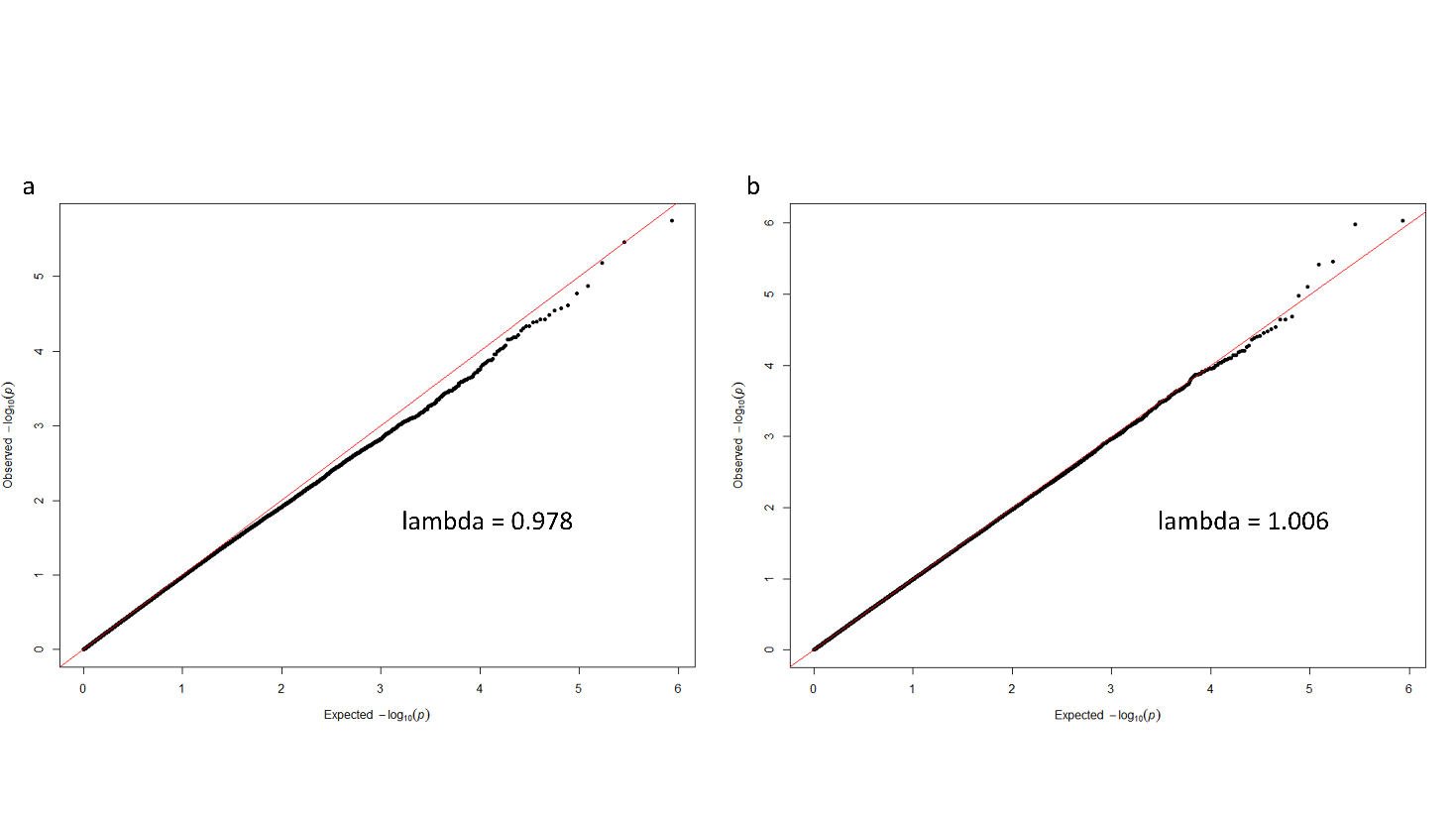


**Supplementary Figure 1.** QQ plots for the model assessing the association between metabolic syndrome and methylation site using the Illumina HumanMethylation450 BeadChip for the overall population. Both models included smoking status, antipsychotic type, CD4T, CD8T, Granulocytes, Monocytes and Natural Killer Cell counts as covariates. Plot a) depicts the QQ plot before performing surrogate variable adjustment (lambda = 0.978) and b) after performing surrogate variable adjustment (lambda = 1.0006).

***Supplementary Table 1. Annotated Top Differentially Methylated CpG Sites (FDR<0.1) Associated with Metabolic Syndrome in the Overall Sample***

| **CpG** | **Gene** | **CpG Type** | **Biological Function** | **Cardiometabolic Reference(s)** |
| --- | --- | --- | --- | --- |
| cg04640913 | Cadherin-like 22 (*CDH22*)* | South Shore | Encodes a calcium-dependent, cell adhesion protein that may play a role in the development of both neuronal and non-neuronal tissues important in diabetes | Bento, 2008;Sugimoto, 1996;Lewis, 2010 |
| cg12501957 | family with sequence similarity 19 (chemokine (C-C motif)-like), member A2  (*FAM19A2*) | Open Sea | Thought to function as brain-specific chemokines that act as regulators of immune and nervous cells | Parsa, 2011 ;Comuzzie, 2012;Kathiresan, 2007 |
| cg05086443 | *CDH5** | South Shore | Classic cadherin from the cadherin superfamily that may play important role in endothelial function by its effects on intercellular junctions | Koga, 2005;Bernard, 2009;Rahimi, 1999 |
| Cg16653173 | Casein kinase 1 (*CSNK1E*) | South Shore | Protein kinase that has the ability to phosphorylate many proteins (including circadian rhythm proteins) and may be involved in DNA replication and repair. Previously associated with schizophrenia and bipolar disorder ([1](#_ENREF_1), [2](#_ENREF_2)) | Levy, 2007;Chang, 2013 |
| cg16656316 | Delta/Notch-like EGF Repeat (*DNER*) | Open Sea | May play a role in Notch signaling pathways which may be involved in intestinal barrier function, beta-cell function and insulin resistance in liver | Deng, 2015;Hanson, 2014 |
| cg06378976 | Transcription Factor EB (*TFEB*) | South Shore | May play critical role in expression of lysosomal and autophagy-related gene transcription | Liu, 2015;Settembre, 2014 |
| cg04457354 | E2F transcription factor 3 (*E2F2*) | Open Sea | Transcription factor binds to protein that regulates expression of gene involved in the cell cycle | Rady, 2013 |
| cg04953503 | *MLPH* | Open Sea | Codes for Rab effector protein involved in melanosome transport | Kasai, 2005 |
| Cg05434957 | Islet autoantigen 1 (*ICA1*) | Island | Protein found in cytosol believe to be involved as autoantigen in type 1 diabetes mellitus | Barkalifa, 2010;Kulkarni, 2015 |
| cg08464505 | ATPase, class VI, type 11A (*ATP11A*) | South Shore | Codes for ATPase membrane protein that requires phosphorylation to transport ions across membrane | Grimsby, 2012 |
| Cg22158175 | Proteosome Subunit, beta type, 8 (*PSMB8*) | North Sea | Proteosome with ATP-dependent proteolytic activity | Deng, 1995 |
| Cg17492940 | protein phosphatase 1, regulatory subunit 12B  (*PPP1R12B*) | Open Sea | Regulates myosin phosphatase activity | Pham, 2012;Geetha, 2012;Grassie, 2011;de las Fuentes, 2012 |
| Cg04033559 | pyruvate dehydrogenase kinase, isozyme 1 (*PDK1*) | Open Sea | Catalyzes the inactivation of pyruvate dehydrogenase and is one of the major enzymes responsible for the regulation of homeostasis of carbohydrate fuels | Hashimoto, 2006;Yamada, 2002;Jeoung, 2015 |

*Supplementary Table 1. Top Differentially Methylated Sites (FDR <0.1) Associated with Metabolic Syndrome Annotated with Biological Function and Previous links to Cardiometabolic Phenotypes in the Literature.* Expanded table 1 from main manuscript including a description of the known or proposed biological function of the associated gene and previous literature references investigating its role in cardiometabolic outcomes (references listed at end of supplementary file).

***Supplementary Table 2. Top 100 Differentially Methylated CpG Sites Associated with Metabolic Syndrome in the Overall Sample***

| **CpG Probe** | **Gene** | **Chromosome** | **Start** | **CpG type** | **Fold Change^a^** | **Raw P-value** | **FDR P-value** |
| --- | --- | --- | --- | --- | --- | --- | --- |
| cg04640913 | CDH22 | chr20 | 44880515 | South Shore | 0.1227 | 9.26E-07 | 0.028975 |
| cg12501957 | FAM19A2 | chr12 | 62629234 | Open Sea | -0.0266 | 1.05E-06 | 0.041984 |
| cg05086443 | CDH5 | chr16 | 66437349 | South Shore | -0.02147 | 3.45E-06 | 0.041984 |
| cg16653173 | CSNK1E | chr22 | 38713453 | South Shore | 0.06746 | 3.83E-06 | 0.044795 |
| cg16656316 | DNER | chr2 | 2.3E+08 | Open Sea | -0.07641 | 7.85E-06 | 0.049115 |
| cg06378976 | TFEB | chr6 | 41703613 | South Shore | 0.135129 | 1.05E-05 | 0.088436 |
| cg04457354 | E2F2 | chr6 | 20447442 | Open Sea | -0.02213 | 2.03E-05 | 0.088436 |
| cg04953503 | MLPH | chr2 | 2.38E+08 | Open Sea | -0.00763 | 2.24E-05 | 0.088436 |
| cg05434957 | ICA1 | chr7 | 8301435 | Island | 0.118456 | 2.26E-05 | 0.092249 |
| cg08464505 | ATP11A | chr13 | 1.13E+08 | South Shore | -0.01129 | 2.87E-05 | 0.092249 |
| cg22158175 | PSMB8 | chr6 | 32809475 | North Shelf | -0.01625 | 3.10E-05 | 0.092249 |
| cg17492940 | PPP1R12B | chr1 | 2.02E+08 | Open Sea | -0.0038 | 3.30E-05 | 0.105488 |
| cg04033559 | PDK1 | chr2 | 1.73E+08 | Open Sea | -0.312 | 3.46E-05 | 0.105488 |
| cg14465344 | LRRC14B | chr5 | 192529 | Island | -0.01049 | 3.81E-05 | 0.202547 |
| cg08658407 | ZNF846 | chr19 | 9903639 | Island | 0.066986 | 3.97E-05 | 0.222297 |
| cg15226102 | ORAOV1 | chr11 | 69469719 | South Shore | -0.04853 | 4.15E-05 | 0.222297 |
| cg01867111 | PRPF40A | chr2 | 1.54E+08 | Island | 0.076 | 4.30E-05 | 0.406614 |
| cg06709089 | SENP3 | chr17 | 7466447 | South Shore | -0.0465 | 5.19E-05 | 0.406614 |
| cg16466303 | TSN3 | chr7 | 47343426 | Open Sea | 0.033508 | 5.45E-05 | 0.66743 |
| cg14405825 | DIP2C | chr10 | 652597 | South Shore | 0.005026 | 6.20E-05 | 0.741374 |
| cg16196713 | BOP1 | chr8 | 1.46E+08 | Island | -0.07864 | 6.20E-05 | 0.935206 |
| cg16466278 | TMEM62 | chr15 | 43429893 | South Shelf | -0.0152 | 6.40E-05 | 0.935206 |
| cg15006869 | FAM19A5 | chr22 | 49141173 | Island | 0.031621 | 6.47E-05 | 0.935206 |
| cg01061590 | PPFIBP1 | chr12 | 27677242 | Open Sea | 0.056139 | 7.09E-05 | 0.935206 |
| cg04486050 | TMEM204 | chr16 | 1579439 | Open Sea | -0.02181 | 7.13E-05 | 0.935206 |
| cg17679350 | MEF2C-AF1 | chr5 | 88299513 | Open Sea | -0.00767 | 7.15E-05 | 0.935206 |
| cg23327011 | MBP | chr18 | 74729021 | Open Sea | -0.00904 | 7.86E-05 | 0.935206 |
| cg03490766 | SMOC2 | chr6 | 1.69E+08 | Open Sea | 0.012267 | 7.88E-05 | 0.935206 |
| cg09957504 | MFHAS1 | chr8 | 8742058 | Open Sea | -0.01671 | 8.15E-05 | 0.935206 |
| cg11849461 | DNAH9 | chr17 | 11547756 | Open Sea | 0.216234 | 8.23E-05 | 0.935206 |
| cg03032053 | MICALL2 | chr7 | 1495311 | North Shelf | -0.02167 | 8.77E-05 | 0.935206 |
| cg01335204 | ZNF445 | chr3 | 44552057 | Open Sea | 0.065205 | 8.93E-05 | 0.935206 |
| cg19546781 | CCL17 | chr16 | 57438715 | Open Sea | 0.012177 | 9.15E-05 | 0.935206 |
| cg16512895 | CACNA1A | chr19 | 13410117 | Island | -0.03407 | 9.29E-05 | 0.935206 |
| cg10294363 | SCNN1A | chr12 | 6480192 | Open Sea | -0.02579 | 9.95E-05 | 0.935206 |
| cg11758127 | PRKCZ | chr1 | 2002884 | North Shelf | -0.01793 | 0.000101 | 0.935206 |
| cg24766553 | FOXR1 | chr11 | 1.19E+08 | Island | -0.06434 | 0.000107 | 0.935206 |
| cg23670630 | FOXR1 | chr11 | 1.19E+08 | Island | -0.1601 | 0.00011 | 0.935206 |
| cg02700891 | TRH | chr3 | 1.3E+08 | Island | 0.27358 | 0.000111 | 0.935206 |
| cg21723689 | GALNT9 | chr12 | 1.33E+08 | Island | -0.00506 | 0.000113 | 0.935206 |
| cg14084826 | SLC45A3 | chr1 | 2.06E+08 | Island | 0.019161 | 0.000115 | 0.935206 |
| cg10461852 | CTBP2 | chr10 | 1.27E+08 | Open Sea | -0.00773 | 0.000115 | 0.935206 |
| cg18832247 | JARID2 | chr6 | 15300007 | Open Sea | -0.06814 | 0.00012 | 0.935206 |
| cg04369291 | IL20RA | chr6 | 1.37E+08 | Island | 0.068484 | 0.000121 | 0.935206 |
| cg05931265 | UBASH3B | chr11 | 1.23E+08 | South Shore | 0.354772 | 0.000122 | 0.935206 |
| cg12470218 | FAF1 | chr1 | 51110218 | Open Sea | -0.00227 | 0.000128 | 0.935206 |
| cg25702335 | CLDN10 | chr13 | 96205897 | South Shore | 0.668686 | 0.000131 | 0.935206 |
| cg09975804 | NOP9 | chr14 | 24769754 | South Shore | -0.045 | 0.000132 | 0.935206 |
| cg21227867 | KCNT1 | chr9 | 1.39E+08 | North Shore | 0.009574 | 0.000133 | 0.935206 |
| cg09376935 | IGHMBP2 | chr11 | 68707886 | Open Sea | -0.00435 | 0.000134 | 0.935206 |
| cg05759269 | CDKN1A | chr6 | 36646253 | Island | 0.079965 | 0.000135 | 0.935206 |
| cg11007153 | MIR4697HG | chr11 | 1.34E+08 | Open Sea | 0.003597 | 0.000143 | 0.935206 |
| cg06657409 | SLC22A6 | chr11 | 62752264 | Open Sea | 0.096722 | 0.000148 | 0.955498 |
| cg17336638 | CDCA7 | chr2 | 1.74E+08 | Open Sea | 0.0048 | 0.000151 | 0.956508 |
| cg06690548 | SLC7A11 | chr4 | 1.39E+08 | Open Sea | 0.033274 | 0.000163 | 0.994709 |
| cg02557934 | CYFIP1 | chr15 | 22893569 | Island | 0.098602 | 0.000173 | 0.994709 |
| cg04843425 | NDRG2 | chr14 | 21503521 | Open Sea | -0.03126 | 0.000187 | 0.994709 |
| cg22288657 | RNMTL1 | chr17 | 688002 | South Shelf | 0.006292 | 0.000188 | 0.994709 |
| cg11043092 | MCPH1 | chr8 | 6287904 | Open Sea | 0.002841 | 0.000188 | 0.994709 |
| cg27000659 | BAG6 | chr6 | 31617530 | North Shelf | -0.01695 | 0.000195 | 0.994709 |
| cg00637144 | DHRS12 | chr13 | 52360268 | Open Sea | -0.04824 | 0.000197 | 0.994709 |
| cg24065957 | ARL15 | chr5 | 53389853 | Open Sea | 0.005268 | 0.000205 | 0.994709 |
| cg27419119 | LRP1 | chr12 | 57559901 | Open Sea | 0.0085 | 0.000217 | 0.994709 |
| cg05522848 | RAD17 | chr5 | 68710359 | North Shore | -0.0227 | 0.000221 | 0.994709 |
| cg27289104 | NEMF | chr14 | 50319271 | North Shore | 0.083298 | 0.000226 | 0.994709 |
| cg09089053 | CNTD2 | chr19 | 40731797 | North Shore | 0.166335 | 0.000228 | 0.994709 |
| cg13901143 | KIAA1524 | chr3 | 1.08E+08 | Island | 0.070267 | 0.000228 | 0.994709 |
| cg04334947 | KATNAL1 | chr13 | 30881232 | Island | 0.05991 | 0.00023 | 0.994709 |
| cg07724674 | HSD11B2 | chr16 | 67465455 | Island | 0.168113 | 0.000232 | 0.994709 |
| cg23841186 | IGFBP6 | chr12 | 53492662 | South Shore | -0.05344 | 0.000235 | 0.994709 |
| cg15618450 | LINC00636 | chr3 | 1.08E+08 | North Shelf | -0.02951 | 0.000241 | 0.994709 |
| cg18267081 | TSSC1 | chr2 | 3241627 | Open Sea | -0.00187 | 0.000243 | 0.994709 |
| cg03945800 | ADCY9 | chr16 | 4165515 | Island | 0.179859 | 0.000248 | 0.994709 |
| cg17758721 | SNRPB | chr20 | 2451208 | Island | 0.113414 | 0.000253 | 0.994709 |
| cg13440795 | MSRA | chr8 | 10210267 | Open Sea | -0.01503 | 0.000258 | 0.994709 |
| cg05715532 | IHH | chr2 | 2.2E+08 | Island | 0.020199 | 0.00026 | 0.994709 |
| cg09895354 | SSPO | chr7 | 1.49E+08 | Open Sea | 0.012423 | 0.000271 | 0.994709 |
| cg17901584 | HP08874 | chr1 | 55353706 | South Shore | 0.201609 | 0.000273 | 0.994709 |
| cg12271981 | SLITRK5 | chr13 | 88326752 | Island | 0.316851 | 0.000277 | 0.994709 |
| cg07563943 | DNAH5 | chr5 | 13843916 | Open Sea | 0.018597 | 0.000289 | 0.994709 |
| cg01881899 | ABCG1 | chr21 | 43652704 | North Shelf | -0.10912 | 0.000291 | 0.994709 |
| cg04528771 | ABHD15 | chr17 | 27893087 | Island | 0.102487 | 0.000299 | 0.994709 |
| cg05734675 | DIP2C | chr10 | 537033 | Open Sea | 0.186306 | 0.000299 | 0.994709 |
| cg04546691 | ROBO2 | chr3 | 77143393 | North Shelf | 0.008586 | 0.000304 | 0.994709 |
| cg20017253 | NOVA2 | chr19 | 46444011 | Island | -0.10355 | 0.000306 | 0.994709 |
| cg15904821 | RASA2 | chr3 | 1.41E+08 | Island | 0.064146 | 0.000309 | 0.994709 |
| cg13580286 | TNFRSF10B | chr8 | 22925391 | North Shore | 0.256883 | 0.000311 | 0.994709 |
| cg17844121 | PPP1R3A | chr7 | 1.14E+08 | Open Sea | 0.010082 | 0.000315 | 0.994709 |
| cg22817421 | DICER1 | chr14 | 95623838 | Island | 0.068556 | 0.000315 | 0.994709 |
| cg06346138 | MST1L | chr1 | 17086071 | Island | 0.23861 | 0.000318 | 0.994709 |
| cg04474049 | CAV1 | chr7 | 1.16E+08 | North Shore | 0.180875 | 0.000319 | 0.994709 |
| cg12740527 | LMO1 | chr11 | 8289974 | Island | 0.07894 | 0.000321 | 0.994709 |
| cg09412310 | C19orf48 | chr19 | 51301333 | Open Sea | 0.007169 | 0.000324 | 0.994709 |
| cg11368182 | SHC2 | chr19 | 436541 | Island | 0.024881 | 0.000325 | 0.994709 |
| cg23272369 | PRR4 | chr12 | 11002071 | Open Sea | -0.01847 | 0.000327 | 0.994709 |
| cg23086982 | MAN2B1 | chr19 | 12757342 | North Shore | -0.00649 | 0.000327 | 0.994709 |
| cg02983043 | APBA2 | chr15 | 29403333 | North Shelf | -0.00997 | 0.000328 | 0.994709 |
| cg22778957 | TAF4 | chr20 | 60639054 | North Shore | -0.00531 | 0.000333 | 0.994709 |
| cg17229421 | FAM175B | chr10 | 1.27E+08 | Open Sea | -0.00423 | 0.000347 | 0.994709 |
| cg18663874 | RAD1 | chr5 | 34915112 | North Shore | 0.080269 | 0.000349 | 0.994709 |

**Supplementary Table 2.** Top 100 CpG results with annotated genes from Linear regression of methylation sites based on metabolic syndrome adjusted for smoking status, antipsychotic type, estimated cell types and batch effects (components estimated using sva package) in the overall sample.

^a^fold change calculated by log2 of the quotient in methylation in subjects with metabolic syndrome compared to subjects without. Positive fold change indicates an increase in methylation (hypermethylation) in the metabolic syndrome group

***Supplementary Table 3. Top 50 Differentially Methylated CpG Islands Associated with Metabolic Syndrome from the Sex-Specific Analysis***

| **Female** | | | | | | **Male** | | | | | |
| --- | --- | --- | --- | --- | --- | --- | --- | --- | --- | --- | --- |
| **Chromosomal Location (Chr:region)** | **Number of CpGs in Island** | **Gene Name** | **Fold Change^a^** | **Raw P-value** | **FDR Corrected P-value** | **Chromosomal Location (Chr:region)** | **Number of CpGs in Island** | **Gene Name** | **Fold Change^a^** | **Raw P-value** | **FDR Corrected P-value** |
| chr3:185000558-185000896 | 33 | MAP3K13 | -0.19629 | 0.000939 | 0.042259 | chr19:46915312-46915802 | 44 | CCDC8 | 0.092997 | 0.0000186 | 0.032546 |
| chr13:20135400-20136041 | 53 | TPTE2 | 0.026733 | 0.001005 | 0.042259 | chr17:40558006-40558274 | 19 | PTRF | -0.12855 | 0.000278 | 0.102145 |
| chr2:176931576-176932663 | 77 | EVX2 | -0.4099 | 0.00106 | 0.0821567 | chr10:49348138-49348676 | 47 | FRMPd2 | 0.461583 | 0.000769 | 0.47624 |
| chr2:11796851-11797236 | 32 | NTSR2 | 0.089307 | 0.001164 | 0.487966 | chr10:134672370-134672604 | 16 | CFAP46 | 0.016048 | 0.000869 | 0.750859 |
| chr17:15820621-15821325 | 56 | ADORA2B | -0.02395 | 0.001332 | 0.779478 | chr3:51740741-51741413 | 68 | GRM2 | -0.06166 | 0.000935 | 0.750859 |
| chr20:4741346-4741945 | 45 | RASSF2 | 0.272469 | 0.001391 | 0.779478 | chr5:135416205-135416475 | 24 | VTRNA2-1 | -0.29739 | 0.001564 | 0.750859 |
| chr19:44507080-44507300 | 19 | ZNF230 | -0.08726 | 0.001501 | 0.779478 | chr5:135415070-135415307 | 18 | VTRNA2-1 | -0.14665 | 0.001573 | 0.750859 |
| chr17:4498422-4498659 | 22 | SMTNL2 | 0.361426 | 0.00169 | 0.779478 | chr9:44401896-44402175 | 31 | LOC103908605 | 0.264213 | 0.001704 | 0.750859 |
| chr10:29698363-29699044 | 71 | SVIL-AS1 | -0.11789 | 0.002143 | 0.779478 | chr4:183729060-183729344 | 24 | TENM3 | -0.16979 | 0.001786 | 0.750859 |
| chr4:83482745-83483091 | 56 | TMEM150C | -0.27966 | 0.002248 | 0.779478 | chr1:154989750-154990234 | 42 | ZBTB7B | -0.1856 | 0.002347 | 0.750859 |
| chr1:3499903-3500470 | 46 | MEGF6 | 0.008998 | 0.002287 | 0.779478 | chr17:79283864-79284131 | 22 | TMEM105 | 0.087905 | 0.002356 | 0.750859 |
| chr16:30596488-30596900 | 41 | ZNF785 | -0.04636 | 0.002808 | 0.779478 | chr9:139640641-139640885 | 21 | LOC100128593 | 0.093682 | 0.002634 | 0.750859 |
| chr8:144635309-144636026 | 59 | GSDMD | 0.363046 | 0.003041 | 0.779478 | chr7:123172733-123173231 | 44 | IQUB | -0.04591 | 0.00322 | 0.750859 |
| chr20:62052908-62053326 | 30 | KCNQ2 | -0.02569 | 0.003332 | 0.779478 | chr15:66332091-66332353 | 32 | MEGF11 | 0.107853 | 0.003431 | 0.750859 |
| chr1:75590818-75591354 | 40 | LHX8 | -0.17968 | 0.003475 | 0.779478 | chr3:126752796-126753021 | 19 | PLXNA1 | 0.013526 | 0.003723 | 0.750859 |
| chr11:63883856-63885182 | 86 | FLRT1 | -0.00121 | 0.003783 | 0.779478 | chr9:72131222-72132174 | 114 | APBA1 | 0.01494 | 0.004151 | 0.750859 |
| chr18:59221452-59221986 | 64 | CDH20 | 0.069244 | 0.00436 | 0.779478 | chr19:48965003-48965792 | 94 | KCNJ14 | 0.013541 | 0.004347 | 0.750859 |
| chr19:46417529-46417745 | 21 | NANOS2 | -0.09061 | 0.004394 | 0.779478 | chr21:43240352-43240586 | 16 | PRDM15 | 0.003871 | 0.004388 | 0.750859 |
| chr15:26915190-26915753 | 52 | GABRB3 | 0.061974 | 0.004561 | 0.779478 | chr19:58861342-58862406 | 99 | A1BG | 0.021117 | 0.00442 | 0.750859 |
| chr14:24779875-24780932 | 108 | LTB4R2 | 0.275357 | 0.004781 | 0.779478 | chr2:39470743-39471208 | 55 | CDKL4 | 0.040399 | 0.00466 | 0.750859 |
| chr17:40935965-40936180 | 17 | WNK4 | -0.46529 | 0.005155 | 0.779478 | chr21:43654847-43655465 | 75 | ABCG1 | 0.020091 | 0.004686 | 0.750859 |
| chr7:27169573-27170638 | 118 | HOXA4 | 0.022324 | 0.005581 | 0.779478 | chr16:561444-561648 | 15 | RAB11FIP3 | -0.03452 | 0.004796 | 0.750859 |
| chr22:45560512-45560783 | 25 | NUP50 | 0.050223 | 0.005677 | 0.779478 | chr17:77997724-77997951 | 23 | TBC1D16 | 0.034673 | 0.00486 | 0.750859 |
| chr12:54763058-54763288 | 18 | ZNF385A | 0.097557 | 0.005698 | 0.779478 | chr19:2900330-2901203 | 98 | ZNF57 | 0.221165 | 0.00512 | 0.750859 |
| chr11:78780677-78781048 | 39 | TENM4 | 0.019623 | 0.005773 | 0.779478 | chr10:48827593-48828126 | 47 | PTPN20B | 0.046542 | 0.005227 | 0.750859 |
| chr5:178156130-178156555 | 42 | ZNF354A | 0.047019 | 0.006133 | 0.779478 | chr2:66662043-66662338 | 23 | MEIS1 | -0.14208 | 0.005816 | 0.750859 |
| chr7:39649254-39649510 | 32 | YAE1D1 | -0.05671 | 0.00676 | 0.779478 | chr19:49004514-49004880 | 29 | LMTK3 | 0.116458 | 0.005849 | 0.750859 |
| chr1:47899662-47900385 | 54 | FOXD2-AS1 | -0.03826 | 0.006935 | 0.779478 | chr10:51225001-51225217 | 18 | PARG | -0.00457 | 0.005869 | 0.750859 |
| chr16:1818553-1818916 | 31 | MAPK8IP3 | 0.014312 | 0.0071 | 0.779478 | chr11:121028573-121028927 | 38 | TECTA | 0.03859 | 0.006657 | 0.750859 |
| chr1:26487808-26488606 | 98 | FAM110D | 0.129441 | 0.00712 | 0.779478 | chr19:35615413-35615665 | 23 | LGI4 | 0.499628 | 0.006723 | 0.750859 |
| chr17:40937259-40937480 | 27 | WNK4 | -0.63955 | 0.007125 | 0.779478 | chr21:42213511-42213946 | 34 | DSCAM | -0.23594 | 0.006727 | 0.750859 |
| chr19:911690-911996 | 28 | R3HDM4 | 0.039439 | 0.007192 | 0.779478 | chr7:143582126-143582610 | 36 | TCAF1 | -0.19553 | 0.007046 | 0.750859 |
| chr1:109824314-109824526 | 16 | PSRC1 | -0.03157 | 0.007287 | 0.779478 | chr21:45560663-45560999 | 29 | C21orf33 | 0.037542 | 0.00717 | 0.750859 |
| chr11:1592499-1592810 | 32 | DUSP8 | -0.11737 | 0.007613 | 0.779478 | chr1:17865864-17866630 | 119 | ARHGEF10L | -0.12676 | 0.007208 | 0.750859 |
| chr4:1063742-1063959 | 17 | RNF212 | 0.009957 | 0.007763 | 0.779478 | chr20:44640289-44641210 | 63 | MMP9 | -0.10036 | 0.007242 | 0.750859 |
| chr1:55246743-55247519 | 88 | TTC22 | 0.013154 | 0.00778 | 0.779478 | chr19:18331047-18331395 | 24 | PDE4C | 0.01196 | 0.007286 | 0.750859 |
| chr7:38350921-38351331 | 38 | TARP | 0.293055 | 0.007829 | 0.779478 | chr8:144403267-144403524 | 22 | TOP1MT | -0.00152 | 0.00729 | 0.750859 |
| chr17:56401729-56402343 | 63 | TSPOAP1 | -0.02882 | 0.007844 | 0.779478 | chr14:70078318-70079077 | 103 | SUSD6 | -0.07636 | 0.007325 | 0.750859 |
| chr12:13152820-13153084 | 21 | HEBP1 | -0.38581 | 0.007875 | 0.779478 | chr16:88502476-88502684 | 16 | ZNF469 | 0.032082 | 0.007788 | 0.750859 |
| chr1:112058185-112058590 | 32 | TMIGD3 | -0.09184 | 0.008069 | 0.779478 | chr17:47645366-47645833 | 32 | LOC100288866 | -0.04223 | 0.007789 | 0.750859 |
| chr12:2339163-2339615 | 42 | CACNA1C | 0.036276 | 0.008348 | 0.779478 | chr12:50349080-50349525 | 45 | AQP2 | -0.13246 | 0.007813 | 0.750859 |
| chr20:259790-260148 | 39 | C20orf96 | 0.029031 | 0.00857 | 0.779478 | chr9:38423948-38424584 | 103 | IGFBPL1 | -0.2667 | 0.007843 | 0.750859 |
| chr1:152487979-152488270 | 27 | CRCT1 | -0.25131 | 0.008699 | 0.779478 | chr14:105251402-105251617 | 19 | AKT1 | 0.072737 | 0.007848 | 0.750859 |
| chr11:61684592-61685216 | 91 | RAB3IL1 | -0.07885 | 0.008722 | 0.779478 | chr5:68628423-68628739 | 35 | CCDC125 | 0.563039 | 0.007966 | 0.750859 |
| chr11:62314762-62315054 | 28 | AHNAK | 0.004427 | 0.008914 | 0.779478 | chr6:143381445-143381918 | 52 | AIG1 | -0.06364 | 0.007989 | 0.750859 |
| chr10:48827593-48828126 | 47 | PTPN20 | 0.108387 | 0.00903 | 0.779478 | chr9:130953520-130954251 | 86 | CIZ1 | -0.08641 | 0.008246 | 0.750859 |
| chr17:25932498-25932712 | 20 | KSR1 | 0.007445 | 0.009061 | 0.779478 | chr9:137980464-137980852 | 31 | OLFM1 | -0.25793 | 0.008269 | 0.750859 |
| chr8:61626646-61627074 | 48 | CHD7 | 0.033408 | 0.009138 | 0.779478 | chr21:46677720-46677924 | 22 | LINC00334 | -0.0253 | 0.008643 | 0.750859 |
| chr11:1774589-1775040 | 35 | CTSD | 0.015407 | 0.009152 | 0.779478 | chr5:43037260-43037520 | 22 | LOC648987 | -0.00478 | 0.008744 | 0.750859 |
| chr17:40936446-40936668 | 19 | WNK4 | -0.39777 | 0.009166 | 0.779478 | chr19:6024735-6024988 | 27 | RFX2 | 0.003619 | 0.009113 | 0.750859 |

**Supplementary Table 3.** Top 50 annotated results from sex-specific linear regression of CpG islands based on metabolic syndrome adjusted for smoking status, antipsychotic type, estimated cell types and batch effects (components estimated using sva package).

^a^fold change calculated by log2 of the quotient in methylation in subjects with metabolic syndrome compared to subjects without. Positive fold change indicates an increase in methylation (hypermethylation) in the metabolic syndrome group

***Supplementary Table 4. Top 10 Enriched Pathways for each Discovery Analysis***

| **Overall Analysis of DNA Methylation and Metabolic Syndrome** | | | |
| --- | --- | --- | --- |
| **Pathway Name** | **FDR P-value** | **Ratio^a^** | **Genes^b^** |
| Wnt/-catenin Signaling | 6.17E-04 | 16/166 | SOX7,LRP5,LRP6,CCND1,CSNK1E,WNT3A,AKT1,JUN,CDH5,SOX6,APC2,SFRP5,PPP2R2C,SOX18,CSNK2B,LRP1 |
| PTEN Signaling | 1.95E-03 | 12/119 | ITGB1,AKT1,RRAS2,FLT1,PIK3R1,CDKN1A,PDGFRA,ITGA5,CSNK2B,INSR,CCND1,PRKCZ |
| Cdc42 Signaling | 3.80E-03 | 12/129 | ITGB1,JUN,MAPK14,PPP1R12B,APC2,MYLPF,PAK2,EXOC2,ITGA5,HLA-F,PRKCZ,HLA-E |
| Adipogenesis pathway | 4.57E-03 | 12/132 | PPARG,CTBP1,SAP18,ZNF423,FOXC2,AKT1,EGR2,HDAC4,TNFRSF1A,CTBP2,GTF2H1,FGF1 |
| Chronic Myeloid Leukemia Signaling | 5.89E-03 | 10/103 | CTBP1,STAT5A,AKT1,RRAS2,HDAC4,PIK3R1,CTBP2,CDKN1A,E2F3,CCND1 |
| Molecular Mechanisms of Cancer | 7.59E-03 | 24/368 | ITGB1,LRP5,PIK3R1,LRP6,ITGA5,E2F3,CCND1,BMP10,PRKCZ,ADCY9,JUN,AKT1,CCND2,MAPK14,WNT3A,RRAS2,SUFU,CDKN1A,PAK2,IHH,CASP8,ARHGEF10,CASP7,LRP1 |
| Ceramide Signaling | 8.91E-03 | 9/93 | AKT1,JUN,RRAS2,TNFRSF1A,PIK3R1,PPP2R2C,ENPP7,PRKCZ,KSR1 |
| Huntington's Disease Signaling | 1.00E-02 | 17/238 | GNG4,HDAC4,PIK3R1,HSPA9,POLR2J,CREB5,NGF,AP2A2,HSPA2,PRKCZ,GNB1,GRM5,JUN,AKT1,TAF4,CASP8,CASP7 |
| Macropinocytosis Signaling | 1.20E-02 | 8/81 | ITGB1,RRAS2,PIK3R1,USP6NL,ITGA5,ACTN4,NGF,PRKCZ |
| CDK5 Signaling | 1.23E-02 | 9/98 | ITGB1,LAMC1,ADCY9,MAPK14,RRAS2,PPP2R2C,PPP1R3A,NGF,CACNA1A |
| **Female Analysis of DNA Methylation and Metabolic Syndrome** | | | |
| Axonal Guidance Signaling | 6.17E-04 | 32/443 | ADAMTS7,UNC5A,NTF4,BDNF,SEMA6B,PDGFC,NFATC1,GNG7,EPHA8,MAP2K2,GNAT1,WNT7B,MKNK1,ADAM19,TUBA3C/TUBA3D,PIK3R2,TUBA3E,WNT5B,EFNA2,NGEF,BMP8A,BMP8B,FGFR2,ADAMTS9,PRKCG,DOCK1,EPHA10,MAG,TUBB8,NFATC2,EPHB3,PIK3CD |
| Ephrin A Signaling | 2.19E-03 | 8/60 | VAV2,EFNA2,EPHA10,NGEF,EPHA8,FGFR2,PIK3CD,PIK3R2 |
| Th2 Pathway | 4.68E-03 | 13/146 | CD247,STAT5A,SOCS3,TNFRSF4,MAF,FGFR2,HLA-DQB1,SPI1,CD40,NFATC2,PIK3CD,PIK3R2,NOTCH1 |
| JAK/Stat Signaling | 4.90E-03 | 9/83 | SOCS3,STAT5A,PIAS4,MAP2K2,CDKN1A,FGFR2,PIK3CD,PIK3R2,CCKBR |
| Pancreatic Adenocarcinoma Signaling | 6.31E-03 | 11/118 | CASP9,RALA,MAP2K2,CDKN1A,BRCA2,FGFR2,PIK3CD,PIK3R2,NOTCH1,PDGFC,PLD1 |
| Maturity Onset Diabetes of Young (MODY) Signaling | 6.76E-03 | 4/20 | ALDOB,CACNA1C,INSR,CACNA1A |
| SAPK/JNK Signaling | 6.76E-03 | 10/103 | MAP4K3,MAP3K10,MAP3K13,MAPK8IP2,FGFR2,PIK3CD,PIK3R2,MAPK8IP3,GNG7,NFATC1 |
| STAT3 Pathway | 7.41E-03 | 8/72 | SOCS3,MAP3K10,MAP2K2,CDKN1A,IGF1R,FGFR2,INSR,TNFRSF11A |
| IL-9 Signaling | 7.59E-03 | 6/45 | SOCS3,STAT5A,FGFR2,BCL3,PIK3CD,PIK3R2 |
| Human Embryonic Stem Cell Pluripotency | 8.71E-03 | 12/140 | NTF4,BDNF,WNT7B,BMP8A,UTF1,BMP8B,FGFR2,PIK3CD,PIK3R2,TCF3,PDGFC,WNT5B |
| **Male Analysis of DNA Methylation and Metabolic Syndrome** | | | |
| FAK Signaling | 1.32E-04 | 13/98 | ITGB1,FGFR3,DOCK1,SRC,AKT1,PAK6,CAPN1,ITGA5,FGFR2,PIK3CD,PIK3R2,ACTG1,EGFR |
| Neuropathic Pain Signaling In Dorsal Horn Neurons | 1.55E-04 | 14/113 | FGFR3,GRM7,SRC,KCNN1,GRM2,KCNN4,GRIN3B,KCNQ2,BDNF,PRKCD,FGFR2,PIK3CD,PIK3R2,PRKCG |
| Reelin Signaling in Neurons | 2.75E-04 | 12/92 | ITGB1,FGFR3,SRC,AKT1,CNR2,ARHGEF16,ITGA5,FGFR2,ARHGEF1,PIK3CD,PIK3R2,FGR |
| NF-KB Activation | 5.89E-04 | 11/86 | ITGB1,FGFR3,AKT1,NFKBIA,PRKCD,ITGA5,FGFR2,IKBKAP,PIK3CD,PIK3R2,PRKCG |
| HER-2 Signaling in Breast Cancer | 7.08E-04 | 11/88 | ITGB1,FGFR3,CCNE1,AKT1,PRKCD,CDKN1A,FGFR2,PIK3CD,PIK3R2,PRKCG,EGFR |
| PTEN Signaling | 8.91E-04 | 13/119 | ITGB1,FGFR3,AKT1,FLT4,CDKN1A,ITGA5,FGFR2,PIK3CD,PIK3R2,INPP5K,FOXG1,INPP5D,EGFR |
| Macropinocytosis Signaling | 1.35E-03 | 10/81 | ITGB1,FGFR3,SRC,PDGFA,PRKCD,ITGA5,FGFR2,PIK3CD,PIK3R2,PRKCG |
| 14-3-3-mediated Signaling | 1.86E-03 | 13/129 | SRC,YAP1,TP73,TUBB4B,FGFR2,PRKCG,FGFR3,AKT1,PRKCD,PIK3CD,GFAP,TUBA3E,PIK3R2 |
| HGF Signaling | 1.91E-03 | 12/114 | ITGB1,FGFR3,DOCK1,AKT1,PRKCD,CDKN1A,ITGA5,FGFR2,PIK3CD,PIK3R2,MAP3K4,PRKCG |
| IL-8 Signaling | 2.14E-03 | 17/196 | SRC,NOX4,FLT4,HBEGF,FGFR2,GNG7,PRKCG,FGFR3,RHOQ,AKT1,PRKCD,CXCL1,PIK3CD,PIK3R2,GNG12,MMP9,EGFR |

**Supplementary Table 4.** The 1000 CpG sites (or CpG islands for sex-specific analysis) with the smallest p-values from the discovery analyses were entered into the Core Analysis module of Ingenuity Pathway Analysis (IPA) software. The top 10 canonical pathways are listed in the table for each analysis along with FDR-correct p-values, ratios and genes.
^a^Ratio refers to the number of molecules (genes) that were matched from the entered data set to the total number of molecules in the pathway
^b^Genes refers to the actual genes from the entered dataset that matched within a given pathway

***CITATIONS FOR SUPPLEMENTARY FILE***

*Barkalifa, R., Y. Yagil and C. Yagil (2010). "Sex-specific genetic dissection of diabetes in a rodent model identifies Ica1 and Ndufa4 as major candidate genes." Physiol Genomics 42(3): 445-455.*

*Bento, J. L., N. D. Palmer, M. Zhong, B. Roh, J. P. Lewis, M. R. Wing, H. Pandya, B. I. Freedman, C. D. Langefeld, S. S. Rich, D. W. Bowden and J. C. Mychaleckyj (2008). "Heterogeneity in gene loci associated with type 2 diabetes on human chromosome 20q13.1." Genomics 92(4): 226-234.*

*Bernard, S., R. Loffroy, A. Serusclat, L. Boussel, E. Bonnefoy, C. Thevenon, M. Rabilloud, D. Revel, P. Moulin and P. Douek (2009). "Increased levels of endothelial microparticles CD144 (VE-Cadherin) positives in type 2 diabetic patients with coronary noncalcified plaques evaluated by multidetector computed tomography (MDCT)." Atherosclerosis 203(2): 429-435.*

*Chang, Y. C., Y. F. Chiu, P. H. Liu, S. W. Hee, T. J. Chang, Y. D. Jiang, W. J. Lee, P. C. Lee, H. Y. Kao, J. J. Hwang and L. M. Chuang (2013). "Genetic variation in the NOC gene is associated with body mass index in Chinese subjects." PLoS One 8(7): e69622.*

*Comuzzie, A. G., S. A. Cole, S. L. Laston, V. S. Voruganti, K. Haack, R. A. Gibbs and N. F. Butte (2012). "Novel genetic loci identified for the pathophysiology of childhood obesity in the Hispanic population." PLoS One 7(12): e51954.*

*de las Fuentes, L., W. Yang, V. G. Davila-Roman and C. Gu (2012). "Pathway-based genome-wide association analysis of coronary heart disease identifies biologically important gene sets." Eur J Hum Genet 20(11): 1168-1173.*

*Deng, G. Y., A. Muir, N. K. Maclaren and J. X. She (1995). "Association of LMP2 and LMP7 genes within the major histocompatibility complex with insulin-dependent diabetes mellitus: population and family studies." Am J Hum Genet 56(2): 528-534.*

*Deng, Z., J. Shen, J. Ye, Q. Shu, J. Zhao, M. Fang and T. Zhang (2015). "Association between single nucleotide polymorphisms of delta/notch-like epidermal growth factor (EGF)-related receptor (DNER) and Delta-like 1 Ligand (DLL 1) with the risk of type 2 diabetes mellitus in a Chinese Han population." Cell Biochem Biophys 71(1): 331-335.*

*Geetha, T., P. Langlais, M. Caruso and Z. Yi (2012). "Protein phosphatase 1 regulatory subunit 12A and catalytic subunit delta, new members in the phosphatidylinositide 3 kinase insulin-signaling pathway." J Endocrinol 214(3): 437-443.*

*Gonzalez, R., S. Gonzalez, E. Villa, M. Ramirez, J. Zavala, R. Armas, J. Contreras, A. Dassori, R. J. Leach and D. Flores (2015). "Identification of circadian gene variants in bipolar disorder in Latino populations." Journal of affective disorders 186: 367-375.*

*Grassie, M. E., L. D. Moffat, M. P. Walsh and J. A. MacDonald (2011). "The myosin phosphatase targeting protein (MYPT) family: a regulated mechanism for achieving substrate specificity of the catalytic subunit of protein phosphatase type 1delta." Arch Biochem Biophys 510(2): 147-159.*

*Grimsby, J. L., B. C. Porneala, J. L. Vassy, Q. Yang, J. C. Florez, J. Dupuis, T. Liu, A. Yesupriya, M. H. Chang, R. M. Ned, N. F. Dowling, M. J. Khoury and J. B. Meigs (2012). "Race-ethnic differences in the association of genetic loci with HbA1c levels and mortality in U.S. adults: the third National Health and Nutrition Examination Survey (NHANES III)." BMC Med Genet 13: 30.*

*Hanson, R. L., Y. L. Muller, S. Kobes, T. Guo, L. Bian, V. Ossowski, K. Wiedrich, J. Sutherland, C. Wiedrich, D. Mahkee, K. Huang, M. Abdussamad, M. Traurig, E. J. Weil, R. G. Nelson, P. H. Bennett, W. C. Knowler, C. Bogardus and L. J. Baier (2014). "A genome-wide association study in American Indians implicates DNER as a susceptibility locus for type 2 diabetes." Diabetes 63(1): 369-376.*

*Hashimoto, N., Y. Kido, T. Uchida, S. Asahara, Y. Shigeyama, T. Matsuda, A. Takeda, D. Tsuchihashi, A. Nishizawa, W. Ogawa, Y. Fujimoto, H. Okamura, K. C. Arden, P. L. Herrera, T. Noda and M. Kasuga (2006). "Ablation of PDK1 in pancreatic beta cells induces diabetes as a result of loss of beta cell mass." Nat Genet 38(5): 589-593.*

*Huang, Y., J. Li, L. Wu, Q. Jin, X. Zhao, J. Li and G. Zhu (2012). "Association between a casein kinase 1 epsilon gene polymorphism and schizophrenia in a Chinese Han population." J Mol Neurosci 47(3): 470-474.*

*Jeoung, N. H. (2015). "Pyruvate Dehydrogenase Kinases: Therapeutic Targets for Diabetes and Cancers." Diabetes Metab J 39(3): 188-197.*

*Kasai, K., M. Ohara-Imaizumi, N. Takahashi, S. Mizutani, S. Zhao, T. Kikuta, H. Kasai, S. Nagamatsu, H. Gomi and T. Izumi (2005). "Rab27a mediates the tight docking of insulin granules onto the plasma membrane during glucose stimulation." J Clin Invest 115(2): 388-396.*

*Kathiresan, S., A. K. Manning, S. Demissie, R. B. D'Agostino, A. Surti, C. Guiducci, L. Gianniny, N. P. Burtt, O. Melander, M. Orho-Melander, D. K. Arnett, G. M. Peloso, J. M. Ordovas and L. A. Cupples (2007). "A genome-wide association study for blood lipid phenotypes in the Framingham Heart Study." BMC Med Genet 8 Suppl 1: S17.*

*Koga, H., S. Sugiyama, K. Kugiyama, K. Watanabe, H. Fukushima, T. Tanaka, T. Sakamoto, M. Yoshimura, H. Jinnouchi and H. Ogawa (2005). "Elevated levels of VE-cadherin-positive endothelial microparticles in patients with type 2 diabetes mellitus and coronary artery disease." J Am Coll Cardiol 45(10): 1622-1630.*

*Kulkarni, H., M. Z. Kos, J. Neary, T. D. Dyer, J. W. Kent, Jr., H. H. Goring, S. A. Cole, A. G. Comuzzie, L. Almasy, M. C. Mahaney, J. E. Curran, J. Blangero and M. A. Carless (2015). "Novel epigenetic determinants of type 2 diabetes in Mexican-American families." Hum Mol Genet 24(18): 5330-5344.*

*Levy, D., M. G. Larson, E. J. Benjamin, C. Newton-Cheh, T. J. Wang, S. J. Hwang, R. S. Vasan and G. F. Mitchell (2007). "Framingham Heart Study 100K Project: genome-wide associations for blood pressure and arterial stiffness." BMC Med Genet 8 Suppl 1: S3.*

*Lewis, J. P., N. D. Palmer, J. B. Ellington, J. Divers, M. C. Ng, L. Lu, C. D. Langefeld, B. I. Freedman and D. W. Bowden (2010). "Analysis of candidate genes on chromosome 20q12-13.1 reveals evidence for BMI mediated association of PREX1 with type 2 diabetes in European Americans." Genomics 96(4): 211-219.*

*Liu, F., Q. Sun, L. Wang, S. Nie and J. Li (2015). "Bioinformatics analysis of abnormal DNA methylation in muscle samples from monozygotic twins discordant for type 2 diabetes." Mol Med Rep 12(1): 351-356.*

*Parsa, A., Y. P. Chang, R. J. Kelly, M. C. Corretti, K. A. Ryan, S. W. Robinson, S. S. Gottlieb, S. L. Kardia, A. R. Shuldiner and S. B. Liggett (2011). "Hypertrophy-associated polymorphisms ascertained in a founder cohort applied to heart failure risk and mortality." Clin Transl Sci 4(1): 17-23.*

*Pham, K., P. Langlais, X. Zhang, A. Chao, M. Zingsheim and Z. Yi (2012). "Insulin-stimulated phosphorylation of protein phosphatase 1 regulatory subunit 12B revealed by HPLC-ESI-MS/MS." Proteome Sci 10(1): 52.*

*Rady, B., Y. Chen, P. Vaca, Q. Wang, Y. Wang, P. Salmon and J. Oberholzer (2013). "Overexpression of E2F3 promotes proliferation of functional human beta cells without induction of apoptosis." Cell Cycle 12(16): 2691-2702.*

*Rahimi, N. and A. Kazlauskas (1999). "A role for cadherin-5 in regulation of vascular endothelial growth factor receptor 2 activity in endothelial cells." Mol Biol Cell 10(10): 3401-3407.*

*Settembre, C. and A. Ballabio (2014). "Lysosome: regulator of lipid degradation pathways." Trends Cell Biol 24(12): 743-750.*

*Sugimoto, K., S. Honda, T. Yamamoto, T. Ueki, M. Monden, A. Kaji, K. Matsumoto and T. Nakamura (1996). "Molecular cloning and characterization of a newly identified member of the cadherin family, PB-cadherin." J Biol Chem 271(19): 11548-11556.*

*Yamada, T., H. Katagiri, T. Asano, M. Tsuru, K. Inukai, H. Ono, T. Kodama, M. Kikuchi and Y. Oka (2002). "Role of PDK1 in insulin-signaling pathway for glucose metabolism in 3T3-L1 adipocytes." Am J Physiol Endocrinol Metab 282(6): E1385-1394.*
